# The role of plant hormones during grafting

**DOI:** 10.1007/s10265-017-0994-5

**Published:** 2017-11-27

**Authors:** Amrit K. Nanda, Charles W. Melnyk

**Affiliations:** Department of Plant Biology, Swedish University of Agricultural Sciences, Almas allé 5, 756 51 Uppsala, Sweden

**Keywords:** Cell division, Cell differentiation, Plant grafting, Phytohormones, Vasculature, Wounding

## Abstract

For millennia, people have cut and joined different plant tissues together through a process known as grafting. By creating a chimeric organism, desirable properties from two plants combine to enhance disease resistance, abiotic stress tolerance, vigour or facilitate the asexual propagation of plants. In addition, grafting has been extremely informative in science for studying and identifying the long-distance movement of molecules. Despite its increasing use in horticulture and science, how plants undertake the process of grafting remains elusive. Here, we discuss specifically the role of eight major plant hormones during the wound healing and vascular formation process, two phenomena involved in grafting. We furthermore present the roles of these hormones during graft formation and highlight knowledge gaps and future areas of interest for the field of grafting biology.

## Introduction

The plant vascular tissue is crucial for transporting water, nutrients, photosynthetic products and signalling molecules throughout the plant. It provides mechanical support, thereby allowing an increase in plant stature and adaptation to various environments. As such, plants have efficient mechanisms to generate vasculature in newly formed organs, to increase vasculature in existing organs, and to heal the vasculature upon wounding or stress. There are two main types of vascular tissues: xylem, made up of tracheary elements, parenchyma cells and fibres; and phloem, made up of sieve elements and parenchyma cells, including companion cells (reviewed by De Rybel et al. 2016; Miyashima et al. 2013; Nieminen et al. 2015). Xylem transports water, minerals and nutrients from roots to shoots, whereas phloem transports photoassimilates from source to sink tissues. Signalling molecules, such as phytohormones, are also transported by these tissues. Vascular tissues come in various patterns depending on plant species, age and organ type. However, in dicotyledonous plants xylem and phloem tissues are always separated by cambium cells and xylem tissue is found towards the central region, whereas phloem tissue develops towards the outer region. The cambium acts as a vascular meristem and during secondary growth divides to produce more xylem and phloem cells, thereby permitting plant stems to increase in diameter (discussed in Spicer and Groover 2010). In the case of monocotyledonous plants, the vascular tissue does not include cambium and these plants rarely undergo secondary growth.

Grafting, consisting of cutting and joining two plants so that they grow together as one, is widely used in horticulture as a method to improve disease resistance, tolerance to abiotic stress, fruit quality and plant size (summarized by Mudge et al. 2009). More recently, researchers have used grafting as a tool to demonstrate the long-distance movement of molecules including phytohormones, proteins and RNAs (summarized by Goldschmidt 2014; Turnbull et al. 2002; Turnbull 2010). Successful grafting requires tissue reunion including forming vascular connections between the two plants. Grafting initially triggers the secretion of pectins from cells at the cut site to adhere the rootstock and scion together. Dedifferentiated stem-cell like tissue, termed callus, then forms at both junctions until the grafted tissues join and plasmodesmata can bridge the connection site. Cambium, cortex and pith cells surrounding the phloem and xylem divide and, together with callus cells, differentiate into vascular tissues and connect the two junctions (Jeffree and Yeoman 1983; Lindsay et al. 1974; Melnyk et al. 2015; Moore 1983; Moore and Walker 1981; Ribeiro et al. 2015). Phloem connections typically precede xylem connections at the graft junction (Melnyk et al. 2015).

Phytohormones regulate every aspect of plant development and responses to biotic and abiotic stresses. Here, we investigate the role of eight major plant hormones during the process of plant grafting. Where insufficient information on their role in grafting is present, we also look to the role of these hormones in wound healing and vascular formation in plants, since it is thought that graft formation includes elements of these processes (Melnyk 2017). The role of salicylic acid was not addressed as it has not yet been implicated in either vascular development or grafting. Through this review, we aim to provide an index of the role of each hormone during vascular formation, wound healing, and grafting, thereby underlining knowledge gaps for future studies.

## Abscisic acid

The phytohormone abscisic acid (ABA) is important for biotic and abiotic stress responses, including drought response, but also plays a role during plant development such as seed maturation, seed germination and the regulation of stomatal aperture (reviewed in Cutler et al. 2010; Finkelstein 2013; Vishwakarma et al. 2017). A few studies have indirectly linked ABA to vascular formation. For instance, secondary growth in *Arabidopsis* induces the expression of several genes containing ABA-inducible *cis*-elements in their promoter region, suggesting they are under the control of ABA signalling (Oh et al. 2003). Moreover, vascular formation and ABA signalling appear to converge on the homeodomain-leucine zipper-type transcription factor (TF), *ARABIDOPSIS THALIANA HOMEOBOX 7* (*ATHB7*), since this gene is specifically expressed in differentiating xylem and is also induced by drought stress and ABA treatment (Hjellström et al. 2003; Söderman et al. 1994; Wenzel et al. 2008). However, more work is needed to fully understand the role of ABA during vascular formation.

Abiotic stresses, including wounding, trigger ABA accumulation (Peña-Cortés et al. 1989). ABA application induces the expression of wound-activated genes in the absence of wounding whereas when ABA-deficient mutants are wounded, wound-activated genes show reduced induction (Peña-Cortés et al. 1989, 1995), suggesting a direct role for ABA in the wound response. Alternatively, ABA accumulation near the wound may be due to desiccation of damaged tissues rather than a direct response to wounding itself (Birkenmeier and Ryan 1998). Further studies are needed to distinguish these two hypotheses. Interestingly, a recent study demonstrated that mutants with reduced ABA synthesis or signalling were more efficient at forming wound-induced callus, suggesting that ABA might have inhibitory aspects towards wound-healing (Ikeuchi et al. 2017). To our knowledge, the role of ABA during grafting has yet to be addressed.

## Auxin

Auxin is the most studied phytohormone and is involved in nearly every developmental process including apical dominance, organ formation and gravitropism (reviewed in Enders and Strader 2015; Teale et al. 2006; Vanneste and Friml 2009; Zhao 2010). Auxin also plays a central role in vascular formation. When applied exogenously to undifferentiated tissues, it promotes the formation of vascular strands (Aloni 1980; Sachs 1981; Wetmore and Rier 1963), whereas when auxin signalling is perturbed, leaf vascular patterns are dramatically altered (Mattsson et al. 1999; Sieburth 1999). Multiple organs, including developing leaves, produce auxin where it is transported by proteins, such as the PIN-FORMED (PIN) proteins, towards the base of the plant (Aloni 2001; Mattsson 2003; Mattsson et al. 1999; Scarpella et al. 2006; Sieburth 1999). The channelling and accumulation of auxin is considered one of the earliest events of vascular differentiation (Donner et al. 2009; Wenzel et al. 2007), most likely through its induction of the auxin response TF *MONOPTEROS* (*MP*). MP directly activates the transcription of a homeodomain-leucine zipper III gene, *ATHB8*, necessary for preprocambial cell specification and the coordination of procambial cell identity. Modulation of auxin levels in specific tissues and cells regulates vascular cell fate. For instance, high auxin levels increase the number of secondary xylem and phloem cells in petunia (Klee et al. 1987), whereas low auxin levels reduce the number of xylem cells in tobacco (Romano et al. 1991). Application of a low amount of auxin differentiates phloem in callus of several plant species, while a high amount induces both phloem and xylem (Aloni 1980).

Auxin is also involved in the wound response, with PIN proteins transporting auxin to the wound site, where it triggers vascular tissue regeneration in *Arabidopsis* stems and pea epicotyl (Mazur et al. 2016; Sauer et al. 2006). For instance, auxin transported basipetally from leaves and buds promotes xylem regeneration following wounding, as removal of these tissues above, but not below, the cut site decreased xylem regeneration of wounded *Coleus blumei* internodes (Jacobs 1952). Likewise, incised *Arabidopsis* stems show an asymmetric auxin accumulation due to a block in basipetal auxin transportation (Asahina et al. 2011). This asymmetry plays an important signalling role during tissue reunion since the *AUXIN RESPONSE FACTOR* (*ARF*) *6* and *8* promote the expression of the NAM, ATAF, and CUC (NAC) TF *ANAC071* above the cut, while inhibiting expression of *RELATED TO AP2 6L* (*RAP2.6L*) (Asahina et al. 2011; Pitaksaringkarn et al. 2014a). In contrast, lower auxin levels below the cut leads to a decrease in *ARF6* and *8* expression, thereby releasing *RAP2.6L* inhibition. *ANAC071* and *RAP2.6L* promote pith cell division and their expression in the top and lower part of the cut site, respectively, is important for successful tissue reunion of incised stems (Asahina et al. 2011). Moreover, *ANAC071* both promotes the expression of and directly binds to the promoters of auxin-inducible *XYLOGLUCAN ENDOTRANSGLUCOSYLASE*/*HYDROLASE* (*XTH*) *19* and *20*, important for pith cell proliferation during tissue reunion (Pitaksaringkarn et al. 2014b). However, the downstream targets of *RAP2.6L* remain unknown. Ethylene and jasmonic acid also regulate *ANAC071* and *RAP2.6L* expression, respectively, and are likely also important for wound healing.

Consistent with auxin’s pivotal role in vascular formation and wound healing, it is perhaps unsurprising that auxin also plays an important role in graft formation. Grafting induces the expression of auxin biosynthesis and signalling genes and exogenous auxin application is often necessary for *in vitro* grafting (Asahina et al. 2011; Chen et al. 2017; Matsuoka et al. 2016; Melnyk et al. 2015; Parkinson and Yeoman 1982; Wang et al. 2014; Yin et al. 2012). Similarly, hypocotyl graft reunion in *Arabidopsis* is inhibited when cotyledons are removed or when cotyledons are treated with an inhibitor of auxin transport (Matsuoka et al. 2016). Cotyledons are an important source of auxin in developing plants (Bhalerao et al. 2002; Katsumi et al. 1969; Procko et al. 2014), and it is likely that cotyledon-derived auxin promotes graft formation in young plants. Interestingly, many *Arabidopsis* mutants perturbed in auxin biosynthesis or transport reconnect their phloem with similar dynamics as wild-type plants (Melnyk et al. 2015). These plants likely successfully graft since auxin levels and auxin transport are not completely abolished. However, some mutants affected in auxin perception or auxin response experience delayed phloem reconnection during hypocotyl grafting (Melnyk et al. 2015), suggesting that how auxin is perceived rather than absolute auxin levels or efficiency of auxin transport may be a determining factor in grafting success. One of the genes important for graft formation, *ABERRANT LATERAL ROOT FORMATION 4* (*ALF4*), acts downstream of auxin and regulates xylem pole pericycle cell division and lateral root formation (Celenza et al. 1995; DiDonato et al. 2004). Xylem pole pericycle cells are important for lateral root formation and *in vitro* callus formation (Atta et al. 2009; Sugimoto et al. 2010) and this cell type, or other meristematic cells in the vascular tissue, might be a key driver of vascular connection during grafting.

## Brassinosteroids

Brassinosteroids (BRs) are steroid hormones involved in cell growth and plant morphogenesis (reviewed in Belkhadir and Jaillais 2015; Saini et al. 2015; Zhu et al. 2013). BRs also promote xylem formation. For instance, inhibition of BR biosynthesis represses tracheary element differentiation in *Zinnia elegans* cells (Iwasaki and Shibaoka 1991) and represses the formation of secondary xylem in cress plants, affecting the phloem to xylem ratio in favour of more phloem (Nagata et al. 2001). Moreover, BR accumulation peaks prior to tracheary element differentiation (Yamamoto et al. 2001). Several *Arabidopsis* mutants affected in BR biosynthesis (*cpd: constitutive photomorphogenesis and dwarfism; dwf7*/*ste1*: *dwarf 7*/*sterol 1*) or response (*bri1: brassinosteroids insensitive 1*) repress xylem formation (Szekeres et al. 1996; Choe et al. 1999; Caño-Delgado et al. 2004). While *BRI1* is expressed ubiquitously in the plant, its homologues *BRL1, 2* and *3* are specifically expressed in the vascular tissues of *Arabidopsis* (Caño-Delgado et al. 2004), further supporting a direct role for BRs in promoting xylem formation.

To our knowledge, a role for BRs during wounding or grafting has not been investigated. However, studies using grafting as a tool to investigate BR transport in pea plants showed that the BR biosynthesis mutant *lkb* (named after the internode length locus *Lk*) grafts successfully (Reid and Ross 1989; Symons 2004). Therefore, BRs are unlikely to be crucial for grafting. BRs interact with different phytohormones in a very wide range of biological processes (reviewed by Saini et al. 2015). For example, during *Arabidopsis* root growth, BR, auxin and cytokinin signalling pathways interact through *BRAVIS RADIX* (*BRX*), a regulator of protophloem differentiation, which is both induced by auxin and slightly repressed by BRs (Mouchel et al. 2006; Scacchi et al. 2010). Considering the potential role of BRs in xylem formation and its interaction with other phytohormones important for grafting, such as auxin, it would be interesting to further investigate the role, if any, of BRs during grafting.

## Cytokinins

Cytokinins (CKs) are adenine-derived phytohormones involved in various aspects of plant development including cell division, lateral root formation and meristem maintenance. Their effects often occur through interaction with the auxin signalling pathway (reviewed in Kieber and Schaller 2014; Osugi and Sakakibara 2015; Schaller et al. 2015). CKs also regulate cambium activity in a dosage-dependent manner (Matsumoto-Kitano et al. 2008). Reducing CK levels inhibits cambial cell divisions and produces thinner stems in *Arabidopsis* and poplar (Matsumoto-Kitano et al. 2008; Nieminen et al. 2008), while increasing CK biosynthesis increases CK signalling and cambium cell division activity in poplar (Immanen et al. 2016). Moreover, loss of function of the *Arabidopsis* CK receptor *CK RESPONSE 1 (CRE1)*/*WOODEN LEG (WOL)*/*ARABIDOPSIS HISTIDINE KINASE 4 (AHK4)* alone, or in combination with *AHK2* and *AHK3*, results in reduced cell file number in root vascular bundles, due to reduced cambial activity (Mähönen et al. 2000; Nishimura et al. 2004). In *Arabidopsis*, CKs mediate auxin transport during vascular formation by regulating the distribution of PIN proteins in developing vascular tissues (Bishopp et al. 2011). In turn, auxin promotes xylem differentiation through the induction of *HISTIDINE PHOSPHOTRANSFER PROTEIN 6* (*AHP6*), a CK signalling inhibitor. Several CK signalling mutants produce extra xylem, while exogenous CK treatments reduce xylem formation (Mähönen et al. 2006; Yokoyama et al. 2006). These results suggest that CKs interact antagonistically with auxin and negatively regulate xylogenesis.

During wounding in *Arabidopsis*, CK biosynthesis genes are upregulated, CK levels increase and CK response is enhanced (Ikeuchi et al. 2017; Iwase et al. 2011). These responses are thought to produce wound-induced callus at the cut surface since *WOUND INDUCED DEDIFFERENTIATION 1* (*WIND1*), a wound-inducible TF, upregulates and promotes callus formation through the activation of CK signalling (Iwase et al. 2011). In addition, CK-deficient mutants produce less callus (Ikeuchi et al. 2017). These results demonstrate the importance of the CK signalling pathway for callus formation in response to wounding.

However, CKs do not appear to be as important for grafting, since several CK biosynthesis and signalling mutants of *Arabidopsis* and poplar graft successfully (Nieminen et al. 2008; Melnyk et al. 2015). Moreover, exogenous application of CKs to explant internodes of several plant species stimulates the formation of vascular strands at the graft union site, but requires the presence of auxin to promote vascular reconnection (Parkinson and Yeoman 1982). Thus, it appears that CKs enhance vascular reconnection during grafting.

## Ethylene

Ethylene is a gaseous hormone that regulates a wide range of processes, including root initiation, fruit ripening, senescence and response to biotic and abiotic stress (reviewed in Lin et al. 2009; Wang et al. 2002). Its mode of action occurs in part through a family of APETALA2/ETHYLENE RESPONSIVE FACTOR (AP2/ERF) TFs that act downstream of the ethylene signalling pathway. These TFs exist in all plant species and are activated in response to multiple stresses or developmental pathways (reviewed in Gu et al. 2017; Licausi et al. 2013).

Ethylene promotes secondary growth in several plant species (Brown and Leopold 1973; Love et al. 2009). In *Arabidopsis*, the receptor kinase *PHLOEM INTERCALATED WITH XYLEM* (*PXY*)/*TRACHEARY ELEMENT DIFFERENTIATION INHIBITOR FACTOR* (*TDIF*) *RECEPTOR* (*TDR*) promotes vascular cell divisions, and loss of this receptor has mild effects due to a compensation mechanism dependent on ethylene (Etchells et al. 2012; Fisher and Turner 2007; Hirakawa et al. 2008). Ethylene regulates the same downstream AP2/ERF TFs as PXY/TDR to promote vascular cell divisions, and mutants over-producing ethylene undergo increased vascular cell divisions. TFs regulated by both ethylene and PXY/TDR are also regulated by *WUSCHEL-RELATED HOMEODOMAIN 4* (*WOX4*), an auxin-inducible TF that interacts with the *PXY*/*TDR* signalling pathway (Etchells et al. 2012; Suer et al. 2011). Therefore, ethylene likely interacts with auxin to promote vascular cell divisions.

Ethylene is associated with abiotic stresses, for instance, wounding triggers ethylene biosynthesis around the site of the wound (Asahina et al. 2011; Watanabe et al. 2001). Incision of *Arabidopsis* stems activates *ANAC071* above the cut, and this activation is partially dependent on ethylene signalling. Suppressing *ANAC071* function inhibited cell division, but not cell elongation, making them incapable of tissue reunion (Asahina et al. 2011). Moreover, wounded *ethylene insensitive 2* (*ein2*) mutants (Alonso et al. 1999) display cell division in the cortex close to the cut, but not in the pith of the stem, resulting in incomplete healing. Interestingly, *ANAC071* expression is also induced by auxin (Matsuoka et al. 2016), suggesting that both hormones interact to promote *ANAC071* expression during tissue reunion.

Transcriptomic analyses of grafted *Arabidopsis* hypocotyls reveal that ethylene biosynthesis genes activate at the graft junction (Yin et al. 2012). However, hypocotyl grafting of mutants enhanced in ethylene signalling (*ctr1: constitutive triple response 1*) or blocked in ethylene response (*ein2, etr1: ethylene receptor 1*) successfully graft (Melnyk et al. 2015). However, mutating *ANAC071* reduced the formation of vascular tissues at the graft junction (Matsuoka et al. 2016). The fact that ethylene signalling is important for tissue reunion in wounded stems but does not appear important in grafted hypocotyls suggests these differences might arise from tissue type, age, or from mechanistic differences between sealing a gap compared to reconnecting the vasculature.

## Gibberellins

Gibberellins (GAs) are diterpene phytohormones with an important role in plant development, particularly in regulating plant growth, as GAs promote cell expansion, cell differentiation and cell proliferation (reviewed in Claeys et al. 2014; Davière and Achard 2013; Yamaguchi 2008). GAs also stimulate xylogenesis in cambium tissue. For instance, GAs accumulate in developing xylem tissue of poplar (Immanen et al. 2016; Israelsson et al. 2005), while expression of three orthologues of the GA receptor *GIBBERELLIN INSENSITIVE DWARF 1* (*GID1*) is highest in the xylem of hybrid aspen (Mauriat and Moritz 2009). Moreover, GA treatment or GA accumulation increases the number and size of xylem fibres of several tree species and tobacco (Biemelt et al. 2004; Dayan et al. 2010; Eriksson et al. 2000), whereas the opposite occurs when GA biosynthesis is inhibited in *Eucalyptus globulus* (Ridoutt et al. 1996).

The role of GAs in wounding is becoming clearer. Cotyledon-derived GAs promote cambium cell division and differentiation in cut cucumber and tomato hypocotyls (Asahina et al. 2002). In grafted *Arabidopsis* hypocotyls, inhibition of GA biosynthesis or signalling suppressed the expansion of cortex cells that seal the graft junction, but did not inhibit cell proliferation in the vascular tissue (Matsuoka et al. 2016). Previous studies have demonstrated that endogenous GA levels decrease following decapitation of pea and tobacco and can be recovered by apical auxin treatments (Ross et al. 2000, 2003; Wolbang and Ross 2001), suggesting that auxin triggers GA biosynthesis or accumulation. In turn, GAs promote auxin transport by modulating the turnover of PIN proteins (Björklund et al. 2007; Willige et al. 2011). Thus, it appears that GAs are important for cell expansion to seal the wound, whereas auxin is important for vascular tissue proliferation and reconnection across the graft junction (Melnyk et al. 2015; Matsuoka et al. 2016).

## Jasmonic acid

Jasmonic acid (JA) and its derivatives are lipid-derived plant hormones primarily known for their activation during biotic and abiotic stress including wounding, but are also involved in plant development such as root development and trichome formation (reviewed in Koo and Howe 2009; León et al. 2001; Santino et al. 2013; Wasternack and Hause 2013). Cambium formation in *Arabidopsis* stems induces JA signalling, suggesting that JAs are implicated in vascular formation (Sehr et al. 2010). Moreover, a recent study demonstrated that JA treatments induce the formation of extra xylem in *Arabidopsis* wild-type roots, but not in mutants affected in JA signalling. It appears that JAs suppress the CK response, allowing more xylem to form (Jang et al. 2017). Further studies are needed to fully understand the role of JA in vascular formation and its interaction with CKs.

JAs accumulate in response to wounding. Incised *Arabidopsis* stems show asymmetric expression of JA signalling genes, with higher expression below the cut compared to above the cut (Asahina et al. 2011). The expression of *RAP2.6L* activates below the cut after incision and methyl jasmonate application, suggesting that it is activated by JA signalling. However, JA signalling also interacts with auxin, as the auxin response factors *ARF6* and *ARF8* suppress the expression of a JA biosynthesis enzyme, *DEFECTIVE IN ANTHER DEHISCENCE 1* (*DAD1*) (Pitaksaringkarn et al. 2014a). In stems, auxin levels are low below a cut due to a block in basipetal auxin transport (Asahina et al. 2011). This reduction could release *ARF6* and *ARF8*-mediated inhibition of *DAD1* expression and result in JA accumulation, thereby inducing *RAP2.6L* expression. These data point to a cross-talk between JA and auxin signalling pathways in the regulation of cell division during tissue reunion. This notion is consistent with other studies demonstrating that JA signalling interacts with auxin biosynthesis and signalling pathways during root development, root stem cell niche maintenance and auxin transport (Chen et al. 2011; Sun et al. 2009, 2011). However, mutants defective in JA signalling or biosynthesis are slightly enhanced in wound-induced callus formation (Ikeuchi et al. 2017), suggesting JAs may inhibit certain aspects of the wound healing response.

Little is known about the role of JAs during grafting. JA-deficient *Arabidopsis* mutants graft successfully (Gasperini et al. 2015), suggesting that JAs are not essential for graft formation. However, *Arabidopsis* hypocotyl grafting induces the expression of JA biosynthesis and signalling genes (Liu et al. 2016; Yin et al. 2012), but whether this represents a broad wound response or instead represents a role for JAs in tissue fusion and vascular formation during grafting remains unknown.

## Strigolactones

Strigolactones (SLs) are carotenoid-derived signalling molecules that regulate plant development and architecture (reviewed in Brewer et al. 2013; Waldie et al. 2014; Waters et al. 2017). SLs also affect cambium cell division since SL-deficient mutants in *Arabidopsis*, pea and *Eucalyptus globulus* have decreased cambium activity whereas SL application increases cambium activity (Agusti et al. 2011). SLs repress PIN expression and PIN protein accumulation in *Arabidopsis* (Crawford et al. 2010), so the enhanced cambium activity mediated by SLs might occur through dampening of auxin transport. However, since *pin1* and *pin3* auxin transport mutants display decreased cambium activity, it has been suggested that SLs can regulate cambium activity in an auxin-independent manner (Agusti et al. 2011).

The role of SLs during the wound response has, to our knowledge, not been addressed. Nevertheless, the role of SLs in cambium activity together with their role in the regulation of auxin transport suggests that SLs might be involved in grafting. However, SL-deficient mutants in various plant species graft successfully (Beveridge et al. 1994, 1996; Drummond et al. 2012; Koltai et al. 2010; Turnbull et al. 2002), emphasizing that SLs are not crucial for grafting success.

## Conclusion

**Fig. 1:**
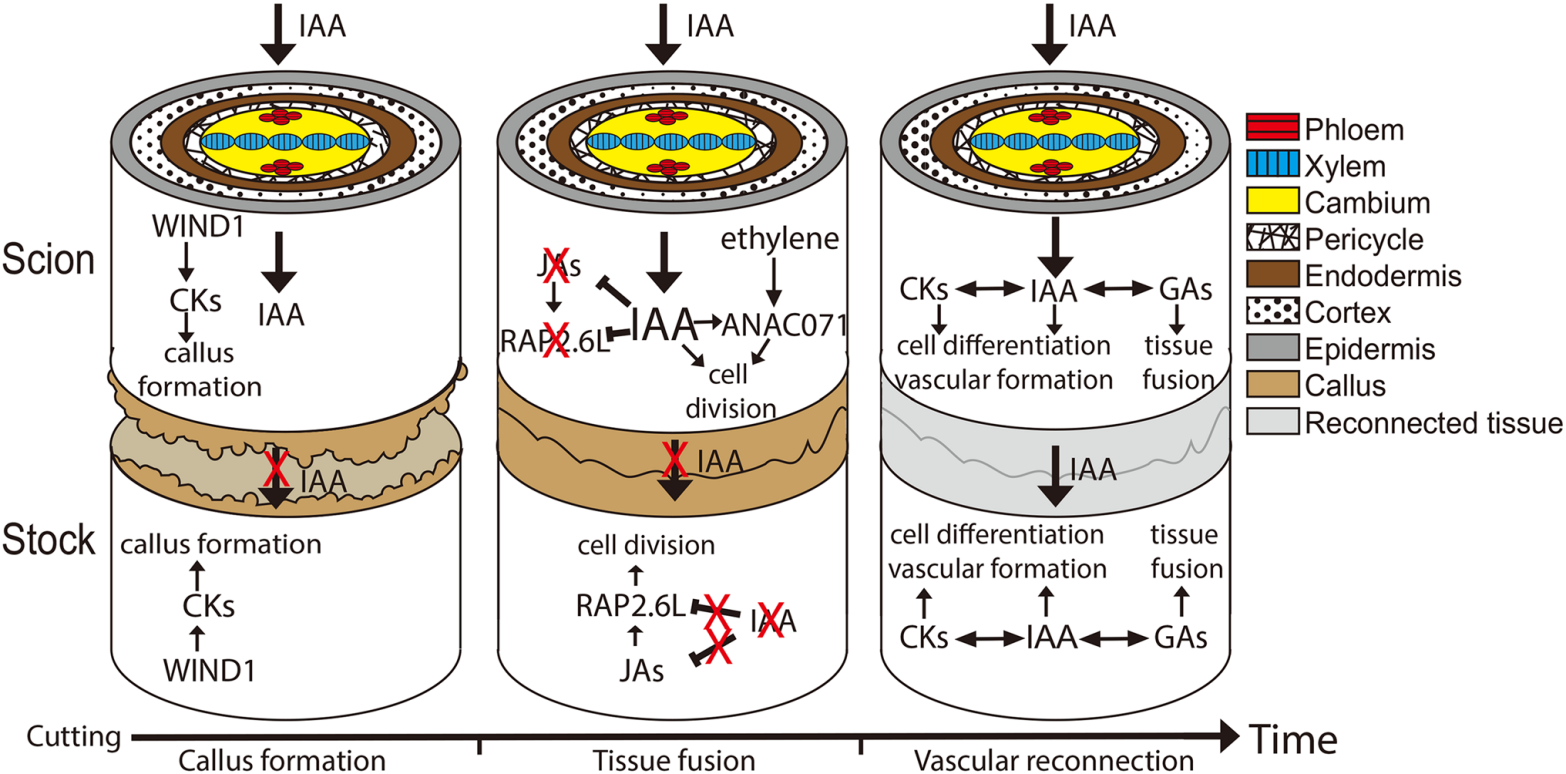
Schematic diagram of the putative grafting process in *Arabidopsis* hypocotyls over time. Following cutting, WIND1 enhances cytokinin response at the graft junctions which induces callus formation. At the same time, auxin, transported basipetally, accumulates above the graft junction and is depleted in the bottom junction since its flow is impaired. Auxin accumulation, in conjunction with ethylene signalling, triggers *ANAC071* expression above the graft junction, while inhibiting jasmonic acid biosynthesis and *RAP2.6L* expression. Below the graft, the decrease in auxin levels releases the suppression of jasmonic acid biosynthesis and *RAP2.6L* expression. *ANAC071* and *RAP2.6L* induced cell division of the vascular tissue both above and below the graft junction, respectively. Auxin, in interaction with gibberellins and cytokinins, promotes cell differentiation, leading to vascular formation and reconnection between both junctions, thereby restoring auxin symmetry. Gibberellins, in interaction with auxin, promote tissue fusion through cell expansion. CKs: cytokinins; IAA: auxin; JAs: jasmonic acids; GAs: gibberellins

The development and differentiation of vascular tissues during plant growth is finely regulated by almost every known phytohormone. Nevertheless, auxin appears to be the primary regulator of vascular cell differentiation and patterning with other hormones interacting with the auxin biosynthesis, transport and/or signalling pathways to fine-tune this process (Fig. 1). Since successful grafting depends on reconnection of the vascular tissues during tissue reunion, it should be unsurprising that the same hormones and genes that regulate vascular formation in developing organs would also regulate vascular tissue regeneration at the graft junction. However, whether vascular reconnection is regulated in an identical manner as vascular formation is unlikely. The cutting process activates wound-responsive hormones and signalling pathways that will likely affect the mechanisms and dynamics of vascular tissue formation and tissue adhesion. Although the role for many phytohormones remains unknown (ABA, salicylic acid) or seems to have little effect (ethylene, JA, SLs, BRs, CKs) on graft formation, it is important to note that many of these conclusions are from qualitative observations that look for short-term success in herbaceous species such as *Arabidopsis* where grafting is robust. Many of these hormones might have more subtle effects on enhancing or suppressing graft formation, and their effects may not be obvious until grafts are more mature. Further in-depth studies are required to fully understand the role of these phytohormones and their contribution to graft formation and to the long-term compatibility and success of the graft in both herbaceous and woody species.
